# Visual search: Heritability and association with general intelligence

**DOI:** 10.1111/gbb.12779

**Published:** 2022-01-19

**Authors:** Patrick D. Quinn, David López Pérez, Daniel P. Kennedy, Sven Bölte, Brian D'Onofrio, Paul Lichtenstein, Terje Falck‐Ytter

**Affiliations:** ^1^ Department of Applied Health Science, School of Public Health Indiana University Bloomington Indiana USA; ^2^ Neurocognitive Development Unit Institute of Psychology, Polish Academy of Sciences Warsaw Poland; ^3^ Deparment of Psychological and Brain Sciences, Cognitive Science Program, Program in Neuroscience Indiana University Bloomington Indiana USA; ^4^ Center of Neurodevelopmental Disorders (KIND), Centre for Psychiatry Research; Department of Women's and Children's Health, Karolinska Institutet & Child and Adolescent Psychiatry Stockholm Health Care Services, Region Stockholm Stockholm Sweden; ^5^ Curtin Autism Research Group, Curtin School of Allied Health Curtin University Perth Western Australia Australia; ^6^ Department of Medical Epidemiology and Biostatistics Karolinska Institutet Stockholm Sweden; ^7^ Development and Neurodiversity Lab, Department of Psychology Uppsala University Uppsala Sweden; ^8^ Swedish Collegium for Advanced Study Uppsala Sweden

**Keywords:** cognition, etiology, evolution, genetics, individual differences

## Abstract

Visual search guides goal‐directed action in humans and many other species, and it has been studied extensively in the past. Yet, no study has investigated the relative contributions of genes and environments to individual differences in visual search performance, or to which extent etiologies are shared with broader cognitive phenotypes. To address this gap, we studied visual search and general intelligence in 156 monozygotic (MZ) and 158 same‐sex dizygotic (DZ) twin pairs. We found that different indexes of visual search performance (response latency and visual search efficiency) were moderately heritable. Phenotypic correlations between visual search and intelligence were small‐to‐moderate, and only a small proportion of the genetic variance in visual search was shared with genetic variance in intelligence. We discuss these findings in the context of the “generalist genes hypothesis” stating that different cognitive functions have a common genetic basis.

## Graphical abstract

We performed a twin study of visual search, which is crucial for goal directed behavior in everyday life. The results indicate that visual search is moderately heritable and largely independent from intelligence.

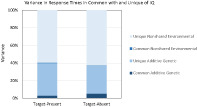

## 1 INTRODUCTION

Every day, we use our eyes to search for specific objects among competing stimuli in the environment, and these search processes influence what we perceive on a moment‐to‐moment basis.^1^, ^2^, ^3^, ^4^ Visual search is tightly linked to the physiological properties of the eye and the visual/oculomotor system. To achieve efficient visual search, the retina must encode a large field of view, and eye movements are needed in order to make potential target areas accessible for high‐resolution (foveal) visual processing. It has been shown that visual search is dependent on parallel detection abilities of the visual system and efficient selection of subsequent fixation locations.^4^ Visual search is likely to have been under evolutionary pressure in many mammalian species,^4^ and represents a phylogenetically old cognitive system compared to other aspects of human cognition.

Given that visual search is a both ubiquitous and special cognitive process, it is valuable to understand its etiology, including its etiological connection to other well‐researched cognitive factors. Some previous studies investigating clinical measures in which visual search is one component indicate that there is substantial unique genetic contributions to visual search.^5^ However, because these measures also reflect other traits (e.g., motor function, eye‐hand coordination, and executive function), one cannot draw strong conclusions about visual search per se based on these findings.

The generalist genes theory predicts a substantial genetic overlap between different areas of cognition, including basic cognitive functions.^6^, ^7^ Face recognition seems to be an exception in the social domain,^8^, ^9^, ^10^ but this view is still dominant for non‐social cognition.^6^, ^7^, ^10^, ^11^ Although twin and GWAS studies indicate that broad cognitive factors such as executive function and intelligence are indeed strongly related at the genetic level,^12^, ^13^, ^14^ recent data suggest that unique genetic variance linked to executive function is important for predicting associations with psychiatric problems.^13^, ^14^ One recent twin study indicated that object recognition was genetically largely uncorrelated with IQ,^10^ suggesting that at least some specific cognitive functions could be more independent than predicted by domain general, generalist theories.

Against this background, we used twin data to assess the heritability of visual search as well as its link to a standardized IQ test battery within a behavioral genetic analytic framework. We leveraged differences in genetic relatedness between monozygotic (sharing virtually all genes) and dizygotic (sharing 50% of segregating alleles, on average) twins to explore genetic and environmental contributions to visual search performance. We fit a series of structural equation models that partitioned inter‐individual variation into genetic variance, shared environmental variance (i.e., environmental sources of twin similarity) and unique environmental variance (which includes random measurement error). By additionally examining associations across multiple traits, the twin framework can be expanded into multivariate designs (e.g., to investigate the association between visual search and IQ).

## 2 MATERIALS AND METHODS

### 2.1 Participants

The participants in the current study were 156 monozygotic (MZ) and 158 same‐sex dizygotic (DZ) twin pairs (final sample, after excluding 35 pairs due to general exclusion criteria (visual or hearing impairments or significant medical conditions), or insufficient visual search data (see below for details). The participants (mean age = 11.12; *SD* = 1.29 years; range = 9.17–14.13) were recruited from a population‐based twin study in Sweden (CATSS^15^) and were living in the larger Stockholm area. Among the monozygotic twin pairs, 69 (44%) were males; among the dizygotic twin pairs, 72 (46%) were males. The study was approved by the Regional Ethics Review Board in Stockholm, written informed consent was obtained from parents, and gift vouchers were given to the children as incentive for participation (~$30 for each child).

The current experiment is part of a larger twin study called iTWIN (a sub sample of the larger CATSS study^15^; see Supplementary Information S1 for power analysis). iTWIN consists of several eye tracking tasks assessing different types of social and non‐social attentional functions, some of which have been reported on previously.^16^, ^17^, ^18^ In the current report, we analyzed only the visual search stimuli in the iTWIN study. Ref. 16 describes phenotypic associations between visual search and various other traits based on the same dataset as we analyze here. In terms of the distribution of SES (education) in the included families, the iTWIN sample is very similar to the larger CATSS study (for details, see Ref. 18), which in turn covers about 70% of all twins in Sweden.^15^ While the coverage of the population is high, recruitment rates tends to be somewhat higher in families with higher socio‐economic status.^19^

### 2.2 Stimuli and procedure

**FIGURE 1:**
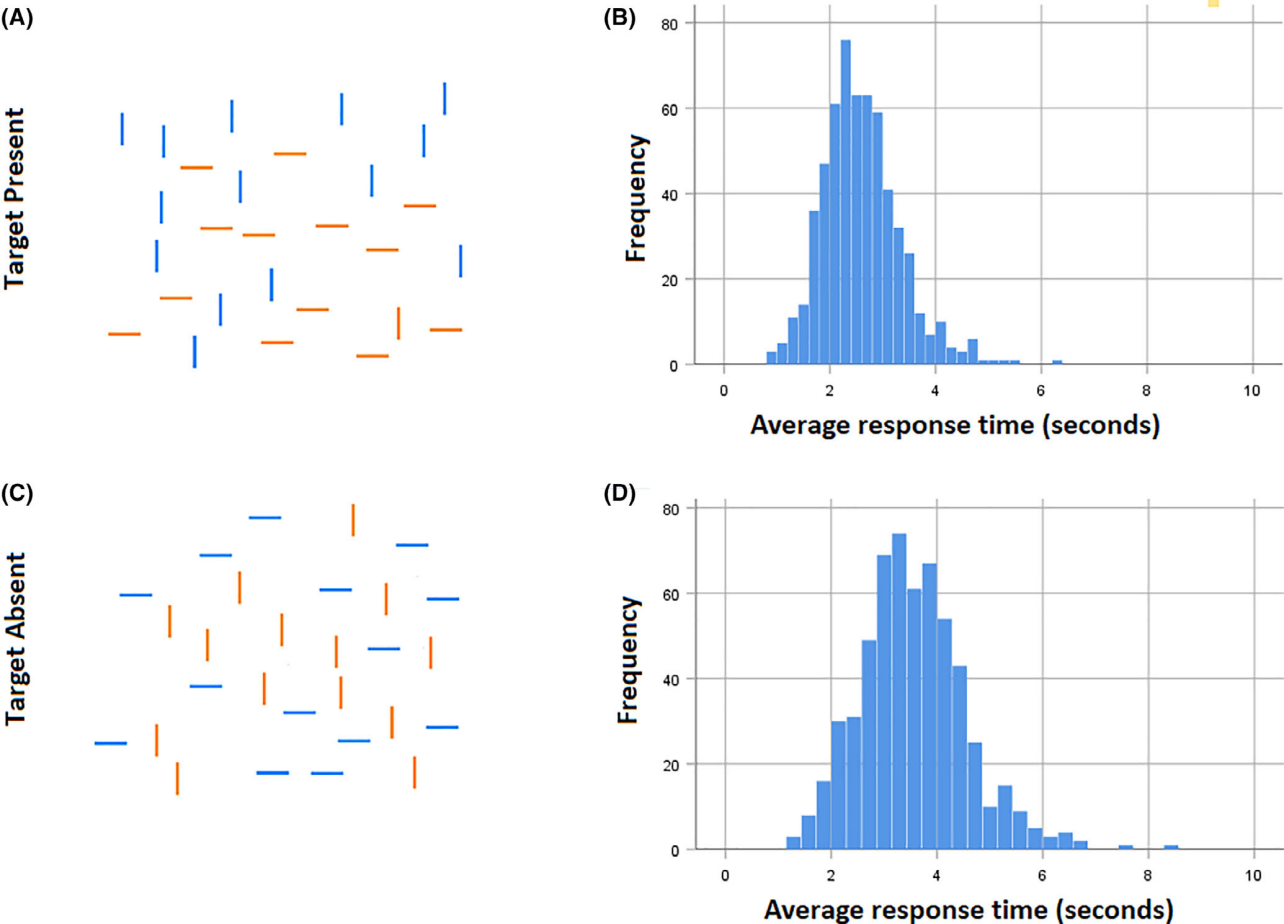
Stimuli (A,B) and corresponding data distributions (C,D). Participants were asked to indicate (through key press) whether a unique target was present in each stimulus. As expected, response times were longer for target absent than for target present trials

The visual search task used rectangular objects that varied in color and orientation.^16^ For examples, see Figure 1. The participants were instructed to determine if one unique target was present in each array, to press with their left index finger if a target was present and with their right index finger if the target was absent, and to do this as quickly as they could (this response time was the main dependent variable in our analyses). On 50% of trials there was a target present (Target Present condition), while on the remaining trials there was no target, only distractors (Target Absent Condition). During 10 training trials, the experimenter ensured that they understood these instructions and were able to follow them.

The visual search task consisted of two search types, conjunction search and feature search, but only the former was analyzed in this study given our focus on individual differences in effortful search and its link to other aspects of cognition. In conjunction search, the target item has a variation of two different feature dimensions (e.g., an orange horizontal rectangle among vertical orange and horizontal blue rectangles; Figure 1). The target item was defined by its uniqueness relative to the distractors on a trial to trial basis (i.e., unless repeated by chance, the target was typically different in each trial—e.g., orange horizontal rectangle in one trial and then blue vertical target in another). Thus, the task was cognitively demanding compared to more simple feature‐based search tasks.^16^

Further, there were three set sizes (8, 18, and 28 elements). In the main analysis, we used set size 28 (1 target, 27 distractors), to include the most difficult condition with the longest response times. Arguably, this approach would provide the most conservative test of etiological independence from general cognitive factors. We used the data from the two other set sizes in additional analyses as described below. Decisions regarding which specific conditions to focus on were done a priori of the data analysis. The number of trials was fully balanced across conditions and set sizes.

Stimuli were presented on a 23″ monitor with a resolution of 1024 × 1280 pixels and responses were registered using a USB keyboard (the stimuli were shown on the standard screen of a Tobii T120 eye tracker which registered eye movements simultaneously with key press). The computer program Tobii Studio (Tobii, Stockholm, Sweden) was used both for presentation and for recording. The CIE coordinates of the stimuli were [0.26, 0.23, 0.98] for blue, [0.49, 0.36, 0.04] for orange and [0.95, 1, 1] for white background. From a distance of 60 cm, the visual angle of the monitor was 29.32 degrees of width and 24.22 degrees of height. The average luminance was 188 cd/m^2^. Participants completed 10 training trials, during which they were trained to hold their index fingers stable on separate keys during the whole experiment, in order to be able to perform the task without looking at their hands/keys. The experimenter did not start the session until they were able to do this effortlessly.

The trial ended when the participant pressed the key, but the experimenter was instructed to prompt the child to press if he or she did not respond within about 10 s (this very rarely happened). The number of unique trials in the main analyses and supplementary sensitivity analyses included in this study were 60 (2 conditions [Absent vs Present] × 3 set sizes × 10 trials for each combination).

### 2.3 Other cognitive assessments

To assess IQ, we administered four subscales from the Wechsler's Intelligence Scale for Children, 4th edition (WISC‐IV),^16^ a gold standard instrument for assessing cognition in children of this age. As in previous research,^10^ we included a non‐verbal subscale (Matrices) and a verbal subscale (Vocabulary). In addition, to control for general factors that were not controlled for in the previous twin study of object recognition,^10^ we also included short term memory (Digit Span) and a speeded perceptual‐motor task (Coding). All of these scales contribute to the total IQ score on the full‐scale version of the WISC‐IV, and in our study, we used the average of them as our measure of overall IQ.

### 2.4 Data reduction and dependent variables

Data was pre‐processed using in‐house scripts written in Matlab 2017a (The MathWorks Inc., Natick, MA, 2017) as in our previous study.^16^ First, trials were included in the analysis if they contained at least 70% of valid gaze samples for both eyes as defined by the Tobii eye tracker output. Next, response times (RTs) were calculated from the onset of the stimuli until a recorded manual key press. Based on visual inspection of the data and assuming that unreasonably fast RTs represented anticipations and unreasonably slow RTs represented attentional lapses, we considered invalid all trials with RTs < 300 ms or > 10,000 ms. Less than 10% of the entire data set for all observers across all tasks was removed by this method.^16^ We included only trials where the participants responded correctly. This meant that the data in the Target Present condition reflect situations where the participant risked false positives only and the data in the Target Absent reflects situations where there was risk for false negative only. This was done to simplify the analysis and presentation.

There are several dependent variables one can extract from the visual search task,^16^ but we capitalized here on the manual response latency because it is an intuitive and easily interpretable measure and because it showed higher correlation with IQ than other measures in our study (we tested the following other variables: the latency to fixate target, the time elapsed between first fixation and key press). For eye movement analysis we used an established fixation identification algorithm^20^ combined with an area of interest approach.^16^ Figure S1 shows the association between response times and latency of eye movements to the target.

We also included a secondary dependent variable: visual search efficiency. This measure is often used in the visual search literature and captures to what extent increasing numbers of distractors come with an added response latency. Search efficiency can be considered a more pure measure of attentional aspects of visual search because it inherently controls for general motor speed and any perceptual differences that are independent of search difficulty.^21^ Previous research has shown that in general, visual search efficiency is more affected by set size during conjunction search than simple feature search. Search efficiency was calculated by subtracting the response time for set size 8 from the response time for set size 28 for all conjunction searches (absent and present together).

### 2.5 Statistical analyses and twin modeling

A previous study of a different experimental task (visual disengagement) within the iTWIN study suggested the current sample size is sufficient to detect both common and unique genetic effects.^18^ The data showed some slight deviations from normality in the current task, but visual inspection did not suggest any invalid extreme values (Figure 1). We therefore included all data, and additional analyses examined the potential impacts of violations of distributional assumptions.

We examined and analyzed twin correlations using Mplus version 8 via the MplusAutomation package in R version 3.5.2,^22^, ^23^, ^24^ with full‐information maximum likelihood estimation to account for missing values at the individual level. That is, in all analyses, we included pairs with visual search data from at least one of the twins for a given condition (*N* = 314 pairs). A total of 584 individuals contributed data for the Target Present condition, 580 for the Target Absent condition, and 581 for IQ). Given the modest sample size, we combined male and female pairs together. To reduce bias due to between‐pairs demographic differences, we analyzed standardized residuals generated by regressing each outcome on age, gender, their interaction, and age squared.

First, we examined univariate models for each outcome independently. These models decompose variance into additive genetic (A), dominance genetic (D), shared environmental (C), and nonshared environmental (E) components.^25^, ^26^ *D* and *C* variance cannot be estimated simultaneously, so we began with ACE models unless monozygotic (MZ) twin correlations were more than twice the magnitude of dizygotic (DZ) twin correlations (in which case we began with ADE models); Figure S2. Second, we fit bivariate ACE or ADE Cholesky decompositions, which estimate the extent to which genetic and environmental influences on one phenotype (e.g., target Absent) are shared with or unique of influences on another (e.g., target present; Figure S3, ^27^). The bivariate models examined two questions. First, bivariate models of the Target Absent and Target Present conditions examined the extent to which genetic and environmental influences are common across differing visual search conditions. Second, bivariate models of visual search and IQ examined the extent to which visual search is genetically and environmentally distinct of general cognitive ability. We compared full models with reduced models using a variety of fit statistics**.** We fit all univariate and bivariate twin models with bootstrapped standard errors (10,000 draws per model). For the full bivariate ADE and ACE decompositions, we increased the maximum number of iterations from 1000 to 10,000 to enable convergence on each bootstrap draw. For some analyses, we additionally modified starting values to facilitate model fitting related to very small parameter estimates. In some cases, this required specifying starting values close to but not exactly 0 (i.e., 0.001).

## 3 RESULTS

### 3.1 Heritability of visual search (response times)

In line with previous research, response times were faster for target present (2599/741 ms) than for target absent trials (mean/*SD* = 3555/1005 ms; see Figure 1 and methods**)**. For the target present condition, variance decomposition models fit the data well (Table S1) and suggested additive genetic but minimal dominance genetic or shared environmental influences, although confidence intervals were wide and included 0 for all (Table 1). C and A could each be individually dropped without statistically significant loss of model fit, but the AE model fit best. In the AE model, additive genetic influences explained 40% of variance (*a* ^2^ = 0.40, 95% CI: [0.24, 0.54]).

**TABLE 1:** Univariate twin correlations and selected univariate twin model parameter estimates

Variable	*n* (twin pairs)		Twin correlations		Model	Univariate twin model estimates^a^		
	MZ	DZ	*r*MZ	*r*DZ		*a* ^2^	*c* ^2^ or *d* ^2^	*e* ^2^
Target‐present RT	156	158	0.41 (0.27, 0.55)	0.19 (0.03, 0.36)	ADE	0.38 (0.00, 0.52)	0.02 (0.00, 0.50)	0.60 (0.45, 0.75)
					ACE	0.40 (0.00, 0.54)	0.00 (0.00, 0.31)	0.60 (0.47, 0.77)
					**AE**	**0.40 (0.24, 0.54)**	**—**	**0.60 (0.46, 0.76)**
Target‐absent RT	155	158	0.41 (0.27, 0.55)	0.05 (−0.13, 0.22)	ADE	0.00 (0.00, 0.00)	0.41 (0.16, 0.55)	0.59 (0.45, 0.76)
					ACE	0.37 (0.19, 0.52)	0.00 (0.00, 0.00)	0.63 (0.48, 0.81)
					**AE**	**0.37 (0.19, 0.52)**	**—**	**0.63 (0.48, 0.81)**
IQ	149	152	0.77 (0.71, 0.84)	0.34 (0.19, 0.49)	ADE	0.69 (0.07, 0.80)	0.05 (0.00, 0.68)	0.25 (0.18, 0.33)
					ACE	0.75 (0.66, 0.82)	0.00 (0.00, 0.00)	0.25 (0.18, 0.34)
					**AE**	**0.75 (0.66, 0.82)**	**—**	**0.25 (0.18, 0.34)**

*Note*: Best‐fitting model is bolded.

^a^ Values are proportions of additive (*a* ^2^) and dominance (*d* ^2^) genetic and shared (*c* ^2^) and non‐shared (*e* ^2^) environmental variance.

For the target absent condition, as shown in Table 1, the genetic variance was attributable to either additive or dominance influences, depending on the model, and was approximately 40%. Table S1 shows that an *AE* model fit no worse than ADE or ACE (best‐fitting model *a* ^2^ = 0.37 [0.19, 0.52]).

Given deviations from normality assumptions, we natural log transformed the visual search response times to examine the sensitivity of the univariate results. We found similar results (Table S2), with modestly more shared environmental variance (point estimate, 5%) for the Target Present condition. Overall, the log‐transformed results supported the main model results and suggested that the data could be used in bivariate models.

### 3.2 Bivariate analysis of target present and target absent conditions

Participants who responded faster in the target present condition were more likely to respond faster in the target absent condition as well (phenotypic *r* = 0.68 [0.64, 0.73]). The bivariate variance decomposition models build upon the cross‐twin, cross‐trait correlation (i.e., the extent to which one twin's phenotype is associated with another phenotype in their co‐twin; *r*MZ = 0.42 [0.31, 0.53]; *r*DZ = 0.13 [−0.01, 0.27]). Fit for the model estimating within‐ and cross‐twin and within‐ and cross‐trait correlations was *χ* ^2^ (17) = 11.05, *p* = 0.85, CFI = 1.00, RMSEA = 0.00.

In bivariate Cholesky decompositions, an AE model fit best, and the residual Target Absent *A* variance not shared with Target Present variance could be dropped without loss of model fit (Table S3). Notably, across all models, there was virtually no residual genetic variance in target absent RT (i.e., entire overlap in genetic influences). This can also be seen in the model‐implied genetic correlations, which estimate the extent of overlap in genetic influences and were at unity in all models. That is, comparing the two conditions, we found that the genetic factors involved were virtually entirely overlapping (e.g., AE model genetic correlation *r*_A_ = 1.00 [0.89, 1.00]). Nonshared environmental variances were also associated across conditions, albeit to a lesser degree (e.g., the nonshared environmental correlation from the AE model was 0.48 [0.32, 0.61]). Complete parameter estimates from the ACE, ADE, and AE models, as well as from the AE model for log‐transformed response times, are shown in Table S4 (see also consistent results in a sensitivity analysis of the set size 18 condition in supplementary results and Tables S5–S7).

### 3.3 Bivariate analysis of visual search and IQ

Next, we examined the extent to which this etiologic pattern reflected general influences on broader cognitive ability (i.e., IQ). As expected, IQ, as operationalized above **(**methods), was highly heritable (best‐fitting [AE] model *a* ^2^ = 0.75 [0.66, 0.82]; Table 1). Individuals with higher IQ scores had modestly faster Target Present (*r* = −0.18 [−0.27, −0.10]) and Target Absent (*r* = −0.17 [−0.26, −0.08]) response times (see Table S8 for correlations with individual IQ subscales).

**FIGURE 2:**
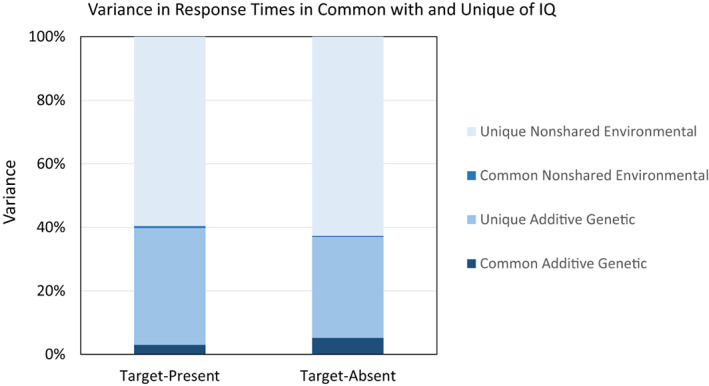
Decomposition of visual search response times into additive genetic (A) and nonshared environmental (E) variance in common with and unique of IQ. Results are presented for the best‐fitting (AE) model

In bivariate Cholesky decompositions of IQ and the target present and target absent conditions, separately, AE models fit best (Table S9). Residual genetic variance in the target present and target absent conditions could not be dropped without loss of model fit, suggesting at least some genetic variance unique of IQ. Genetic correlations between IQ and target present and target absent response times were generally moderate in magnitude (best‐fitting model *r*_A_ = −0.28 [−0.46, −0.08] and *r*_A_ = −0.37 [−0.58, −0.20], respectively; see Table S10, which also reports path coefficients for full and reduced models). Notably, this result implies that most of the genetic influences on visual search (approximately 92% and 86% of the genetic variances, respectively) were independent of those on IQ, as shown in the best‐fitting model variance decomposition (Figure 2). In full ADE decompositions, though, we note that genetic correlations were larger but quite imprecise. That is, overall, we found support for considerable additive genetic (and nonshared environmental) distinction of visual search from IQ.

### 3.4 Visual search efficiency

We repeated this bivariate analysis with our secondary outcome of visual search efficiency. In line with the result for response times, twin correlations for visual search efficiency were consistent with moderate heritability (Table S11). Further, the phenotypic correlation with IQ was very low for this measure (*r* = −0.09 [−0.18, −0.002]). Finally, bivariate Cholesky decompositions showed that, similar to the response time measures, (a) an AE model fit best (Table S12), (b) residual genetic variance in visual search efficiency could not be excluded without loss of model fit, and, (c) genetic factors influencing visual search efficiency were only moderately correlated with those for IQ (best‐fitting model *r*_A_ = −0.37 [−0.96, −0.12]; Table S13).

## 4 DISCUSSION

This study indicates that individual differences in visual search are moderately heritable. This result was found for both the raw response times during difficult conjunction search, and for visual search efficiency (i.e., to what extent visual search slows down with more distractors present). We found virtually no evidence of shared environmental influences and substantial influence of the non‐shared environment (which at least in part reflects error of measurement).

Visual search shared only a small proportion of variance with IQ at the phenotypic level. In particular, visual search efficiency, which controls for general factors like motor speed, shared only around 1% of the phenotypic variance with IQ. Bivariate twin analyses indicated that the genetic factors associated with visual search were largely independent from those underlying IQ (Figure 2; see also Tables S10 and S13). Previous research has shown that socio‐cognitive functions (face recognition) are independent from IQ,^8^, ^9^, ^10^ and the current study indicates a similar largely dissociable genetic structure for visual search.

The conjunction search task used in the current study is cognitively demanding compared to feature based visual search, where the target tends to “pop out” with minimal effort. Previous research indicates that conjunction search recruits a network of frontoparietal areas, including posterior parietal cortex, intraparietal sulcus, frontal eye field, and supplementary motor area/supplementary eye field,^28^, ^29^, ^30^, ^31^, ^32^ and the unique genetic factors associated with visual search in our study could be linked to individual differences in this network. In contrast, global brain processes like overall neuronal plasticity, dendritic complexity, myelination and speed of nerve conduction have been proposed to explain how generalist genes may achieve their widespread effects.^6^

It is notable that we included in our composite IQ measure the subtest “coding” which has visual search as one central component. Indeed, small‐to‐moderate phenotypic correlations confirmed that visual search response time was most strongly associated with this subscale, while the associations with other subscales like matrices and vocabulary were weaker (Table S8). Against this background, it is particularly striking that the genetic overlap between visual search and IQ was minimal in our study.

The observed heritability of visual search of around 40% (Table 1) is lower than the heritability of many higher order cognitive functions (e.g., those captured by IQ scales)^11^ but in line with the heritability of gaze patterns during free exploration of visual scenes at the same age,^17^ see also Ref. 33. Given that the outcome of visual search processes constrain people's perceptions (and consequently, their actions) on a moment‐to‐moment basis, these findings contribute to a new understanding of how genetics may continuously shape an individual's environment via eye movement selection (for a discussion, see Ref. 17).

When one searches for a certain object (e.g., a screw in the toolbox) there are two likely outcomes: you conclude you have found it (and pick it up), or you somehow decide that it is not there (and look elsewhere). These scenarios involve different forms of risks (false positives versus false negatives), and deciding when to terminate a search in the two scenarios calls upon different cognitive processes.^34^ By applying multivariate twin modeling analysis to these two conditions (present, absent), we mapped out how these distinct decision making processes may or may not be linked etiologically. We found that there was a high correlation between the genetic factors involved in the two conditions, suggesting that despite their differences,^34^ highly correlated (or identical) genetic factors govern the neural processes that underlie these types of decisions.

The iTWIN study is one of the largest eye tracking studies of twins published so far. Nevertheless, its sample size was relatively small for multivariate twin modeling, and replication in a larger twin sample assessed for visual search and IQ would strengthen inferences (see power analysis in Supplementary Information S1). The implications of this limitation are two‐fold. First, it reduced the precision of our findings, yielding wider confidence intervals for, for example, genetic correlations. Thus, further research is needed to estimate these associations more precisely. Second, the sample size rendered full bivariate models difficult to fit. Thus, we were unable to differentiate additive from dominance genetic influences, meaning that these influences may be best understood as genetic in the broad sense rather than additive genetic in particular. The same was true for genetic and shared environmental variance for the Target Present condition in the univariate model, although there appeared to be minimal shared environmental variance overall. We also did not test for gender differences. Moreover, we specified different starting values in order to fit some models with very small parameter estimates (e.g., residual target absent genetic variance in the bivariate decomposition), suggesting that some very small parameter estimates (and their precision) should be interpreted with caution. However, we report full and reduced model results in the interest of completeness. Another potential limitation is the generalizability of the results to other age groups. While individual differences in cognitive abilities are generally stable from around 10 years of age,^35^ we do not know how the association (or lack thereof) would generalize to younger samples.

The generalist genes hypothesis applies to cognition in a broad sense, including intelligence as well as basic cognitive processes.^6^ Nevertheless, previous studies of the genetic architecture of (nonsocial) cognition have been biased toward cognitive tasks included in standardized (neuro) psychological tests.^7^, ^36^, ^37^ Except for the aforementioned study of object recognition^10^ and some tasks linked to executive functions (e.g., anti‐saccade task^38^), to our knowledge, experimental tasks of (non‐social) lower level cognitive functions and their association with intelligence have not been reliably assessed in large samples. The current findings highlight the importance of studying a wider range of “higher” and “lower” cognitive functions in order to understand the zones of etiological convergence and divergence at a broader scale.

## CONFLICT OF INTEREST

The authors declare no conflicts of interest.

## Supporting information


**Figure S1** Manual response times is highly predictable from the latency of eye movements to the target.
**Figure S2**. Univariate ACE and ADE models for Target Present RT.
**Figure S3**. Example ADE Cholesky decomposition.
**Table S1**. Univariate Visual Search Model Fit Comparisons.
**Table S2**. Selected Univariate Model Parameter Estimates and Fit Comparisons with Natural Log Transformation.
**Table S3**. Bivariate Visual Search Model Fit Comparisons.
**Table S4**. Bivariate Visual Search Model Results.
**Table S5**. Descriptives and Twin Correlations for Set Size 18 Condition among Included Participants.
**Table S6**. Bivariate Visual Search Model Fit Comparisons for Set Size 18 Condition among Included Participants.
**Table S7**. Bivariate Visual Search Model Results for Set Size 18 Condition among Included Participants.
**Table S8**. Phenotypic Correlations between Visual Search and IQ Subscales.
**Table S9**. Bivariate Visual Search and IQ Model Fit Comparisons..
**Table S10**. Bivariate Visual Search and IQ Model Results.
**Table S11**. Descriptives and Twin Correlations for Visual Search Efficiency among Included Participants.
**Table S12**. Bivariate Visual Search Efficiency and IQ Model Fit Comparisons.
**Table S13** Bivariate Visual Search Efficiency and IQ Model Results.

## Data Availability

The data that support the findings of this study are available on request from the corresponding author (TFY). Transfer of data will require a data processor agreement according to EU legislation (GDPR). The data are not publicly available due to them containing information that could compromise research participant privacy/consent.
