# Supplementary material for: Visual search: Heritability and association with general intelligence
Source: Genes Brain Behav. 2022 Jan 19;21(2):e12779. doi: 10.1111/gbb.12779 (PMC9744476; doi:10.1111/gbb.12779)
Supplement: Supplementary file 1 — Figure S1 Manual response times is highly predictable from the latency of eye movements to the target. Figure S2. Univariate ACE and ADE models for Target Present RT. Figure S3. Example ADE Cholesky decomposition. Table S1. Univariate Visual Search Model Fit Comparisons. Table S2. Selected Univariate Model Parameter Estimates and Fit Comparisons with Natural Log Transformation. Table S3. Bivariate Visual Search Model Fit Comparisons. Table S4. Bivariate Visual Search Model Results. Table S5. Descriptives and Twin Correlations for Set Size 18 Condition among Included Participants. Table S6. Bivariate Visual Search Model Fit Comparisons for Set Size 18 Condition among Included Participants. Table S7. Bivariate Visual Search Model Results for Set Size 18 Condition among Included Participants. Table S8. Phenotypic Correlations between Visual Search and IQ Subscales. Table S9. Bivariate Visual Search and IQ Model Fit Comparisons.. Table S10. Bivariate Visual Search and IQ Model Results. Table S11. Descriptives and Twin Correlations for Visual Search Efficiency among Included Participants. Table S12. Bivariate Visual Search Efficiency and IQ Model Fit Comparisons. Table S13 Bivariate Visual Search Efficiency and IQ Model Results. [file GBB-21-e12779-s001.docx]

**Supporting Information**

**Table of Contents**

[**Power analysis** 2](#_Toc88057296)

[**Supplementary Results** 3](#_Toc88057297)

[**Supplementary Figures** 4](#_Toc88057298)

[Supplementary Figure 1. Manual response times is highly predictable from the latency of eye movements to the target 4](#_Toc88057299)

[Supplementary Figure 2. Univariate ACE and ADE models for Target Present RT 5](#_Toc88057300)

[Genetic covariance constraints are MZ/DZ. 5](#_Toc88057301)

[Supplementary Figure 3. Example ADE Cholesky decomposition 6](#_Toc88057302)

[**Univariate Model Fit Comparisons** 7](#_Toc88057303)

[Supplementary Table 1. Univariate Visual Search Model Fit Comparisons 7](#_Toc88057304)

[**Univariate Sensitivity Analyses** 8](#_Toc88057305)

[Supplementary Table 2. Selected Univariate Model Parameter Estimates and Fit Comparisons with Natural Log Transformation 8](#_Toc88057306)

[**Bivariate Visual Search Model Fit Comparisons and Results** 9](#_Toc88057307)

[Supplementary Table 3. Bivariate Visual Search Model Fit Comparisons 9](#_Toc88057308)

[Supplementary Table 4. Bivariate Visual Search Model Results 10](#_Toc88057309)

[**Bivariate Sensitivity Analyses** 11](#_Toc88057310)

[Supplementary Table 5. Descriptives and Twin Correlations for Set Size 18 Condition among Included Participants 11](#_Toc88057311)

[Supplementary Table 6. Bivariate Visual Search Model Fit Comparisons for Set Size 18 Condition among Included Participants 11](#_Toc88057312)

[Supplementary Table 7. Bivariate Visual Search Model Results for Set Size 18 Condition among Included Participants 12](#_Toc88057313)

[**Bivariate Visual Search and IQ Model Fit Comparisons and Results** 13](#_Toc88057314)

[Supplementary Table 8. Phenotypic Correlations between Visual Search and IQ Subscales 13](#_Toc88057315)

[Supplementary Table 9. Bivariate Visual Search and IQ Model Fit Comparisons 14](#_Toc88057316)

[Supplementary Table 10. Bivariate Visual Search and IQ Model Results 15](#_Toc88057317)

[Supplementary Table 11. Descriptives and Twin Correlations for Visual Search Efficiency among Included Participants 16](#_Toc88057318)

[Supplementary Table 12. Bivariate Visual Search Efficiency and IQ Model Fit Comparisons 16](#_Toc88057319)

[Supplementary Table 13 Bivariate Visual Search Efficiency and IQ Model Results 17](#_Toc88057320)

## **Power analysis**

The current paper describes results that were part of a larger project (iTWIN), which consists of several eye tracking experiments and other types of assessments, several of which have been published on previously (see main text). We conducted a general, broad scope power analysis prior to data collection in iTWIN, assuming a final N of 450 twin pairs (in reality, due to recruitment difficulties, the final N was somewhat lower: 314 pairs in the final sample in this analysis). The a priori power analysis showed that we would have nearly 100% power to detect a heritability of 40% and a shared environmental effect of 40% for a phenotype. We would have 97% power to detect effects of this magnitude when estimating boys and girls separately (presuming an equal number of boys and girls). We would have nearly 100% power to detect a significant genetic contribution to a correlation between two measures, assuming that the heritability of the two variables are 40%, that the shared environment explains 40% of the variation in the two variables, and that the phenotypic correlation is r = 0.40 and to the same degree mediated by genetic and shared environmental effects.  We would have 88% power to detect effects of this magnitude when estimating boys and girls separately (presuming an equal number of boys and girls).

Against this background we reasoned that the current set of analyses were meaningful to conduct, although we noted that power was limited and independent replication would be important for future studies.

## **Supplementary Results**

**Sensitivity analysis (set size 18):** As a test of the robustness of the genetic overlap between the Target Present and Target Absent conditions, we repeated the bivariate analysis using a different visual search condition (set size 18 conjunction search). The results are displayed in **Supplementary Tables 5-7** below. First, twin correlations were similar as for our primary analyses (of set size 28 conjunction search) for both Target Present and Target Absent conditions. Secondly, as for set size 28, there was a substantial phenotypic association (*r* = .63 [.58, .68]), and an *AE* model best fit the bivariate data. In the bivariate *AE* model, the residual Target Absent genetic variance, although not null, could be dropped without loss of model fit. Across all models, genetic correlations were at least .88. That is, results were largely consistent across set sizes 28 and 18. Genetic factors influencing Target Present RTs appear to be largely the same as those that affect Target Absent RTs, and this finding was robust across full and reduced models and across set sizes.

## **Supplementary Figures**

**
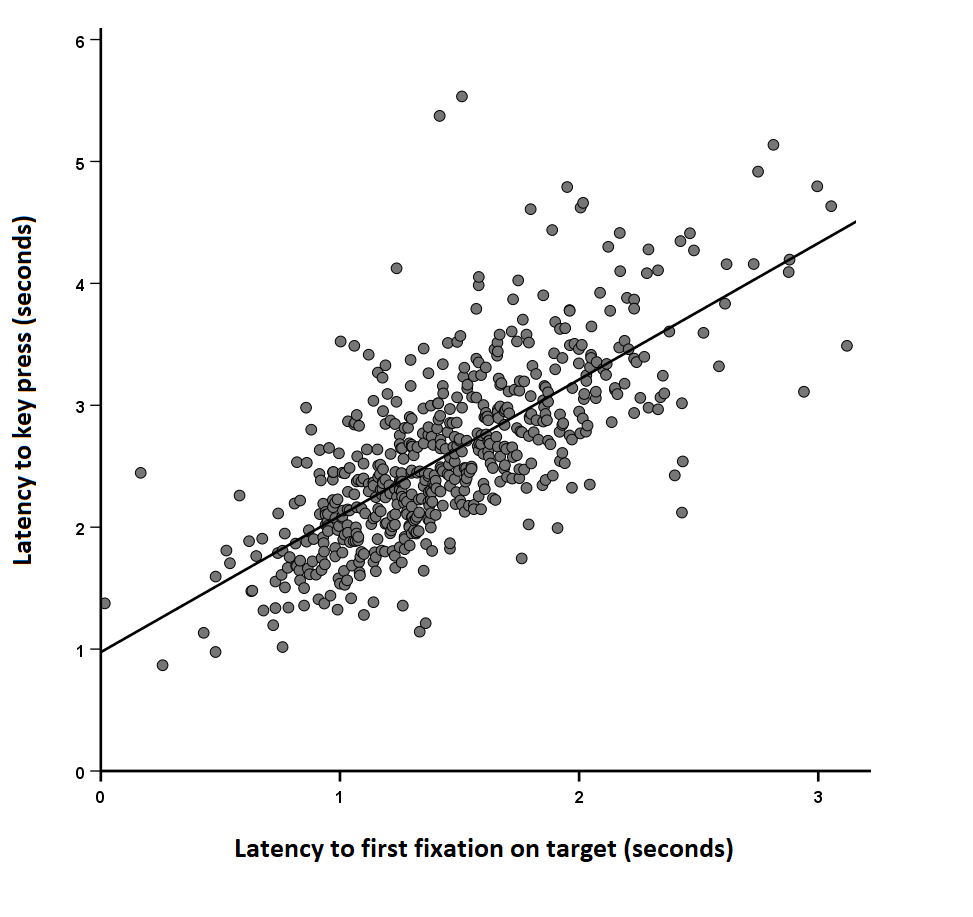
**

Supplementary Figure 1. Manual response times is highly predictable from the latency of eye movements to the target*.* Graph shows data for Target Present condition, set size 28.


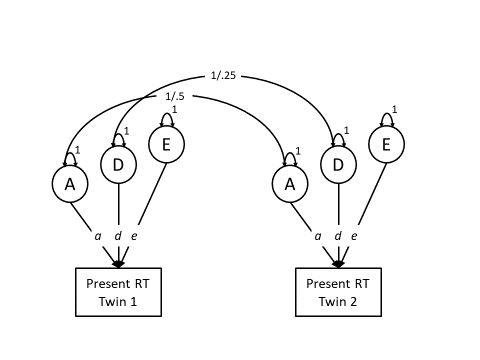

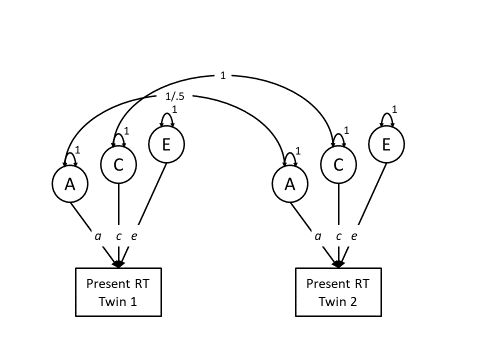


#### Supplementary Figure 2. Univariate ACE and ADE models for Target Present RT

#### Genetic covariance constraints are MZ/DZ.


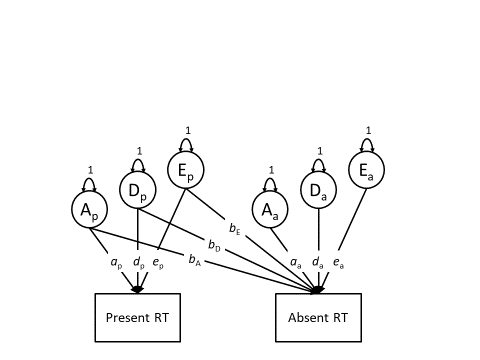


#### Supplementary Figure 3. Example ADE Cholesky decomposition

1 twin per pair shown.

## **Univariate Model Fit Comparisons**

#### Supplementary Table 1. Univariate Visual Search Model Fit Comparisons

| **Variable** | **Model** | **AIC** | **BIC** | **CFI** | **RMSEA** | **χ^2^ (*df*)** | **Comparison Model^a^** | **Δχ^2^ (*df*)** |
| --- | --- | --- | --- | --- | --- | --- | --- | --- |
| Target-Present RT | *ADE* | 1636.32 | 1651.32 | 1.00 | .00 | 2.08 (6) | -- |  |
|  | *ACE* | 1636.33 | 1651.33 | 1.00 | .00 | 2.09 (6) | -- |  |
|  | ***AE*** | **1634.33** | **1645.58** | **1.00** | **.00** | **2.09 (7)** | ***ADE*** | **0.004 (1)** |
|  |  |  |  |  |  |  | ***ACE*** | **0.00 (1)** |
|  | *CE* | 1637.98 | 1649.22 | 1.00 | .00 | 5.73 (7) | *ACE* | 3.65 (1) |
|  | *E* | 1661.24 | 1668.74 | .16 | .14 | 31.00* (8) | *AE* | 28.91* (1) |
| Target-Absent RT | *ADE* | 1630.40 | 1645.39 | 1.00 | .00 | 4.96 (6) | -- |  |
|  | *ACE* | 1632.97 | 1647.96 | .93 | .04 | 7.53 (6) | -- |  |
|  | ***AE*** | **1630.97** | **1642.21** | **.98** | **.02** | **7.53 (7)** | ***ADE*** | **2.57 (1)** |
|  |  |  |  |  |  |  | ***ACE*** | **0.00 (1)** |
|  | *CE* | 1638.28 | 1649.52 | .64 | .09 | 14.83* (7) | *ACE* | 7.31* (1) |
|  | *E* | 1649.91 | 1657.40 | .07 | .13 | 28.47* (8) | *AE* | 20.94* (1) |

*Note*. Bolded model was chosen (best-fitting) reduced model.

^a^ Comparison model is for χ^2^ difference test of nested models.

* *p* < .05 for χ^2^ and Δχ^2^.

## **Univariate Sensitivity Analyses**

#### Supplementary Table 2. Selected Univariate Model Parameter Estimates and Fit Comparisons with Natural Log Transformation

| **Variable** | **Model** | ***a*^2^**  **(95% CI)** | ***c*^2^ or *d*^2^**  **(95% CI)** | ***e*^2^**  **(95% CI)** | **AIC** | **BIC** | **CFI** | **RMSEA** | **χ^2^ (*df*)** | **Comparison Model^a^** | **Δχ^2^ (*df*)** |
| --- | --- | --- | --- | --- | --- | --- | --- | --- | --- | --- | --- |
| Target-Present RT | *ADE* | .34 (.00, .49) | .00 (.00, .30) | .67 (.50, .82) | 1644.06 | 1659.06 | 1.00 | .00 | 4.47 (6) |  |  |
|  | *ACE* | .28 (.00, .48) | .05 (.00, .35) | .67 (.51, .83) | 1644.00 | 1659.00 | 1.00 | .00 | 4.41 (6) | -- |  |
|  | ***AE*** | **.34 (.18, .50)** | **--** | **.67 (.51, .82)** | **1642.06** | **1653.31** | **1.00** | **.00** | **4.47 (7)** | ***ADE*** | **0.00 (1)** |
|  |  |  |  |  |  |  |  |  |  | ***ACE*** | **0.06 (1)** |
| Target-Absent RT | *ADE* | .00 (.00, .00) | .42 (.24, .56) | .58 (.44, .75) | 1627.49 | 1642.47 | 1.00 | .00 | 2.38 (6) | -- |  |
|  | *ACE* | .38 (.21, .52) | .00 (.00, .00) | .62 (.48, .79) | 1630.46 | 1645.45 | 1.00 | .00 | 5.35 (6) | -- |  |
|  | ***AE*** | **.38 (.21, .52)** | **--** | **.62 (.48, .79)** | **1628.46** | **1639.70** | **1.00** | **.00** | **5.35 (7)** | ***ADE*** | **2.97 (1)** |
|  |  |  |  |  |  |  |  |  |  | ***ACE*** | **0.00 (1)** |

*Note*. Target-present *r*MZ = .35 (.20, .50); *r*DZ = .17 (.01, .34). Target-absent *r*MZ = .43 (.29, .57); *r*DZ = .04 (-.13, .22). Bolded model was chosen (**Supplementary Table 1**).

^a^ Comparison model is for χ^2^ difference test of nested models.

* *p* < .05 for χ^2^ and Δχ^2^.

## **Bivariate Visual Search Model Fit Comparisons and Results**

#### Supplementary Table 3. Bivariate Visual Search Model Fit Comparisons

| **Model** | **AIC** | **BIC** | **CFI** | **RMSEA** | **χ^2^ (*df*)** | **Comparison Model^a^** | **Δχ^2^ (*df*)** |
| --- | --- | --- | --- | --- | --- | --- | --- |
| 0. *ACE* | 2941.46 | 2982.71 | 1.00 | .00 | 12.98 (17) | -- |  |
| 1. *ADE* | 2940.47 | 2981.72 | 1.00 | .00 | 11.99 (17) | -- |  |
| **2. *AE*** | **2935.46** | **2965.46** | **1.00** | **.00** | **12.98 (20)** | **0** | 0.00 (3) |
|  |  |  |  |  |  | **1** | **0.99 (3)** |
| **2a. Drop A_a_** | **2933.46** | **2959.71** | **1.00** | **.00** | **12.98 (21)** | **2** | **0.00 (1)** |
| 2b. Drop *b*_A_ | 2969.53 | 2992.03 | .92 | .09 | 51.04* (22) | 2a | 38.07* (1) |

*Note*. Bolded models are best-fitting models.

^a^ Comparison model is for χ^2^ difference test of nested models.

* *p* < .05 for χ^2^ and Δχ^2^.

#### Supplementary Table 4. Bivariate Visual Search Model Results

|  | ***ACE* Model** | ***ADE* Model** | ***AE* Model** | ***AE* Model (ln)** |
| --- | --- | --- | --- | --- |
| ***Unstandardized Parameter Estimates*** | | | |  |
| Target-Present RT | |  |  |  |
| *a*_p_ | 0.64 (0.34, 0.77) | 0.39 (0.00, 0.74) | 0.64 (0.48, 0.78) | 0.59 (0.44, 0.72) |
| *d*_p_ | -- | 0.53 (0.00, 0.75) | -- | -- |
| *c*_p_ | 0.001 (-0.42, 0.46) | -- | -- | -- |
| *e*_p_ | 0.77 (0.67, 0.87) | 0.76 (0.65, 0.85) | 0.77 (0.67, 0.87) | 0.81 (0.69, 0.91) |
| Regression | |  |  |  |
| *b*_A_ | 0.63 (0.34, 0.77) | 0.36 (-0.09, 0.76) | 0.63 (0.43, 0.79) | 0.60 (0.40, 0.73) |
| *b*_D_ | -- | 0.54 (0.00, 0.75) | -- | -- |
| *b*_C_ | 0.00 (-0.47, 0.42) | -- | -- | -- |
| *b*_E_ | 0.38 (0.24, 0.54) | 0.36 (0.21, 0.51) | 0.38 (0.24, 0.53) | 0.45 (0.30, 0.60) |
| Residual Target-Absent RT | |  |  |  |
| *a*_a_ | 0.00 (-0.14, 0.001) | -0.001 (-0.19, 0.004) | 0.00 (-0.29, 0.00) | 0.00 (-0.16, 0.32) |
| *d*_a_ | -- | 0.00 (-0.001, 0.001) | -- | -- |
| *c*_a_ | 0.001 (-0.001, 0.04) | -- | -- | -- |
| *e*_a_ | 0.70 (0.62, 0.76) | 0.70 (0.61, 0.75) | 0.70 (0.62, 0.76) | 0.69 (0.60, 0.74) |
| ***Implied Variance Correlations*** | | | |  |
| *r*_A_ | 1.00 (.95, 1.00) | 1.00 (-1.00, 1.00) | 1.00 (.89, 1.00) | 1.00 (.85, 1.00) |
| *r*_D_ | -- | 1.00 (-.999, 1.00) | -- | -- |
| *r*_C_ | .04 (-1.00, 1.00) | -- | -- | -- |
| *r*_E_ | .48 (.33, .62) | .46 (.30, .60) | .48 (.32, .61) | .55 (.42, .67) |

*Note*. Values are estimate (95% CI). Correlations estimated as, for example, *b*_A_ / √ (*a*_a_^2^ + *b*_A_^2^).

## **Bivariate Sensitivity Analyses**

#### Supplementary Table 5. Descriptives and Twin Correlations for Set Size 18 Condition among Included Participants

| **Variable** | ***n (*Individuals)** | **Observed Range** | ***M*** | ***SD*** | ***n* (Twin Pairs)** | | **Twin Correlations** | |
| --- | --- | --- | --- | --- | --- | --- | --- | --- |
|  |  |  |  |  | **MZ** | **DZ** | ***r*MZ** | ***r*DZ** |
| Target-Present RT | 581 | 906-5375 | 2467 | 644 | 156 | 158 | .34 (.18, .49) | .03 (-.13, .20) |
| Target-Absent RT | 582 | 1010-6749 | 3147 | 841 | 156 | 158 | .49 (.36, .62) | .13 (-.03, .30) |

*Note*. Parenthetical values are 95% confidence intervals; unit for latencies is milliseconds.

#### Supplementary Table 6. Bivariate Visual Search Model Fit Comparisons for Set Size 18 Condition among Included Participants

| **Model** | **AIC** | **BIC** | **CFI** | **RMSEA** | **χ^2^ (*df*)** | **Comparison Model^a^** | **Δχ^2^ (*df*)** |
| --- | --- | --- | --- | --- | --- | --- | --- |
| 0. *ACE* | 2996.02 | 3037.27 | 1.00 | .00 | 13.88 (17) | -- |  |
| 1. *ADE* | 2993.46 | 3034.71 | 1.00 | .00 | 11.32 (17) | -- |  |
| **2. *AE*** | **2990.02** | **3020.02** | **1.00** | **.00** | **13.88 (20)** | **0** | 0.00 (3) |
|  |  |  |  |  |  | **1** | **2.56 (3)** |
| **2a. Drop A_a_** | **2989.38** | **3015.62** | **1.00** | **.00** | **15.24 (21)** | **2** | **1.35 (1)** |
| 2b. Drop *b*_A_ | 3018.83 | 3041.33 | .92 | .09 | 46.69* (22) | 2a | 31.45* (1) |

*Note*. Bolded models are best-fitting models. Cross-twin, cross-trait correlations, *r*MZ = .34 (.23, .46); *r*DZ = .09 (-.05, .23). Model fit for cross-twin, cross-trait correlations: χ^2^ (17) = 10.21, *p* = .89, CFI = 1.00, RMSEA = .00.

^a^ Comparison model is for χ^2^ difference test of nested models.

* *p* < .05 for χ^2^ and Δχ^2^.

#### Supplementary Table 7. Bivariate Visual Search Model Results for Set Size 18 Condition among Included Participants

|  | ***ACE* Model** | ***ADE* Model** | ***AE* Model** |
| --- | --- | --- | --- |
| ***Unstandardized Parameter Estimates*** | | | |
| Target-Present RT | |  |  |
| *a*_p_ | 0.54 (0.34, 0.66) | 0.08 (-0.06, 0.59) | 0.54 (0.37, 0.67) |
| *d*_p_ | -- | 0.56 (0.00, 0.66) | -- |
| *c*_p_ | 0.001 (0.00, 0.32) | -- | -- |
| *e*_p_ | 0.84 (0.75, 0.93) | 0.83 (0.73, 0.92) | 0.84 (0.75, 0.93) |
| Regression | |  |  |
| *b*_A_ | 0.60 (0.36, 0.79) | 0.11 (0.00, 0.70) | 0.60 (0.40, 0.79) |
| *b*_D_ | -- | 0.60 (-0.13, 0.78) | -- |
| *b*_C_ | 0.001 (0.00, 0.37) | -- | -- |
| *b*_E_ | 0.37 (0.25, 0.49) | 0.35 (0.23, 0.47) | 0.37 (0.25, 0.49) |
| Residual Target-Absent RT | |  |  |
| *a*_a_ | 0.29 (0.00, 0.43) | 0.00 (-0.02, 0.002) | 0.29 (0.00, 0.44) |
| *d*_a_ | -- | 0.33 (-0.002, 0.45) | -- |
| *c*_a_ | 0.001 (0.00, 0.002) | -- | -- |
| *e*_a_ | 0.65 (0.57, 0.72) | 0.63 (0.55, 0.71) | 0.65 (0.57, 0.72) |
| ***Implied Variance Correlations*** | | | |
| *r*_A_ | .90 (.72, 1.00) | 1.00 (-.999, 1.00) | .90 (.73, 1.00) |
| *r*_D_ | -- | .88 (-1.00, 1.00) | -- |
| *r*_C_ | .80 (-.997, 1.00) | -- | -- |
| *r*_E_ | .49 (.36, .61) | .48 (.34, .61) | .49 (.36, .61) |

*Note*. Values are estimate (95% CI). Correlations estimated as, for example, *b*_A_ / √ (*a*_a_^2^ + *b*_A_^2^).

## **Bivariate Visual Search and IQ Model Fit Comparisons and Results**

#### Supplementary Table 8. Phenotypic Correlations between Visual Search and IQ Subscales

|  | Efficiency | Target Present RT | Target Absent RT | Digit Span | Coding | Vocabulary | Matrices |
| --- | --- | --- | --- | --- | --- | --- | --- |
| Visual Search |  |  |  |  |  |  |  |
| Efficiency | 1 |  |  |  |  |  |  |
| Target Present RT | .67 (.61, .72) | 1 |  |  |  |  |  |
| Target Absent RT | .76 (.71, .80) | .68 (.63, .74) | 1 |  |  |  |  |
| IQ Subtests |  |  |  |  |  |  |  |
| Digit Span | -.06 (-.15, .02) | -.09 (-.17, -.01) | -.11 (-.19, -.02) | 1 |  |  |  |
| Coding | -.16 (-.24, -.08) | -.24 (-.32, -.16) | -.26 (-.33, -.19) | .22 (.13, .30) | 1 |  |  |
| Vocabulary | -.01 (-.10, .08) | -.07 (-.16, .03) | -.03 (-.13, .07) | .26 (.17, .35) | .18 (.08, .28) | 1 |  |
| Matrices | -.01 (-.10, .08) | -.09 (-.18, -.01) | -.05 (-.13, .04) | .29 (.22, .37) | .22 (.13, .31) | .33 (.24, .42) | 1 |

Note. Values are phenotypic correlation (95% confidence interval), estimated in M*plus* and adjusted for non-independence of twin pairs (*n* individuals = 627). Confidence interval for phenotypic association among visual search outcomes varies slightly from value reported elsewhere because of differences in estimation models.

#### Supplementary Table 9. Bivariate Visual Search and IQ Model Fit Comparisons

| **Variable** | **Model** | **AIC** | **BIC** | **CFI** | **RMSEA** | **χ^2^ (*df*)** | **Comparison Model^a^** | **Δχ^2^ (*df*)** |
| --- | --- | --- | --- | --- | --- | --- | --- | --- |
| IQ and Target-Present RT^b^ | 0. *ACE* | 3142.40 | 3183.64 | .999 | .01 | 17.18 (17) | -- |  |
|  | 1. *ADE* | 3142.47 | 3183.72 | .999 | .01 | 17.25 (17) | -- |  |
|  | **2. *AE*** | **3136.95** | **3166.95** | **1.00** | **.00** | **17.73 (20)** | **0** | **0.55 (3)** |
|  |  |  |  |  |  |  | **1** | **0.48 (3)** |
|  | 2a. Drop A_p_ | 3161.73 | 3187.98 | .87 | .08 | 44.51* (21) | 2 | 26.78* (1) |
|  | 2b. Drop *b*_A_ | 3165.35 | 3187.85 | .85 | .09 | 50.13* (22) | 2a | 5.62* (1) |
| IQ and Target-Absent RT^c^ | 0. *ACE* | 3142.00 | 3183.25 | .99 | .02 | 18.00 (17) | -- |  |
|  | 1. *ADE* | 3139.52 | 3180.76 | 1.00 | .00 | 15.51 (17) | -- |  |
|  | 2. *AE* | **3136.00** | **3166.00** | **1.00** | **.00** | **18.00 (20)** | 0 | 0.00 (3) |
|  |  |  |  |  |  |  | 1 | **2.48 (3)** |
|  | 2a. Drop A_a_ | 3150.35 | 3176.60 | .92 | .06 | 34.35* (21) | 2 | **16.35* (1)** |
|  | 2b. Drop *b*_A_ | 3163.71 | 3186.21 | .84 | .09 | 49.70* (22) | 2a | 15.36* (1) |

*Note*. Bolded models were best-fitting models.

^a^ Comparison model is for χ^2^ difference test of nested models.

^b^ Cross-twin, cross-trait correlations, *r*MZ = -.16 (-.26, -.06); *r*DZ = -.02 (-.14, .11). Model fit: χ^2^ (17) = 16.68, *p* = .48, CFI = 1.00, RMSEA = .00.

^c^ Cross-twin, cross-trait correlations, *r*MZ = -.20 (-.30, -.09); *r*DZ = -.08 (-.19, .04). Model fit: χ^2^ (17) = 15.06, *p* = .59, CFI = 1.00, RMSEA = .00.

* *p* < .05 for χ^2^ and Δχ^2^.

#### Supplementary Table 10. Bivariate Visual Search and IQ Model Results

|  | **Target-Present RT and IQ** | | | |  | **Target-Absent RT and IQ** | | |
| --- | --- | --- | --- | --- | --- | --- | --- | --- |
|  | ***ACE* Model** | ***ADE* Model** | | ***AE* Model** |  | ***ACE* Model** | ***ADE* Model** | ***AE* Model** |
| ***Unstandardized Parameter Estimates*** | | | | | | | | |
| IQ | |  | |  |  |  |  |  |
| *a*_IQ_ | 0.84 (0.65, 0.93) | 0.78 (0.36, 0.91) | | 0.86 (0.77, 0.94) |  | 0.86 (0.66, 0.94) | 0.84 (0.38, 0.92) | 0.86 (0.77, 0.95) |
| *d*_IQ_ | -- | 0.35 (-0.13, 0.76) | | -- |  | -- | 0.20 (-0.46, 0.76) | -- |
| *c*_IQ_ | 0.22 (-0.20, 0.54) | -- | | -- |  | 0.001 (0.00, 0.53) | -- | -- |
| *e*_IQ_ | 0.50 (0.43, 0.58) | 0.50 (0.43, 0.57) | | 0.50 (0.43, 0.57) |  | 0.50 (0.43, 0.58) | 0.50 (0.43, 0.57) | 0.50 (0.43, 0.57) |
| Regression | |  | |  |  |  |  |  |
| *b*_A_ | -0.25 (-0.46, -0.04) | -0.02 (-0.34, 0.41) | | -0.17 (-0.30, -0.05) |  | -0.23 (-0.42, -0.11) | -0.13 (-0.45, 0.23) | -0.23 (-0.34, -0.11) |
| *b*_D_ | -- | -0.38 (-0.69, 0.09) | | -- |  | -- | -0.44 (-0.72, 0.001) | -- |
| *b*_C_ | 0.24 (-0.11, 0.58) | -- | | -- |  | 0.001 (-0.15, 0.29) | -- | -- |
| *b*_E_ | -0.07 (-0.20, 0.07) | -0.07 (-0.19, 0.07) | | -0.08 (-0.21, 0.06) |  | 0.05 (-0.08, 0.18) | 0.06 (-0.07, 0.18) | 0.05 (-0.08, 0.18) |
| Residual Target-Present or -Absent RT | | |  | |  |  |  |  |
| *a*_p_ or *a*_a_ | 0.52 (0.00, 0.69) | 0.51 (0.00, 0.70) | | 0.61 (0.45, 0.73) |  | 0.57 (0.21, 0.70) | 0.00 (-0.53, 0.49) | 0.57 (0.38, 0.70) |
| *d*_p_ or *d*_a_ | -- | 0.00 (-0.29, 0.42) | | -- |  | -- | 0.44 (-0.001, 0.67) | -- |
| *c*_p_ or *c*_a_ | 0.00 (-0.01, 0.39) | -- | | -- |  | 0.001 (0.00, 0.001) | -- | -- |
| *e*_p_ or *e*_a_ | 0.78 (0.67, 0.87) | 0.77 (0.66, 0.86) | | 0.77 (0.67, 0.86) |  | 0.79 (0.69, 0.89) | 0.77 (0.67, 0.87) | 0.79 (0.69, 0.89) |
| ***Implied Variance Correlations*** | | | | | | | | |
| *r*_A_ | -.43 (-1.00, -.08) | -.05 (-1.00, 1.00) | | -.28 (-.46, -.08) |  | -.37 (-.88, -.18) | -1.00 (-1.00, 1.00) | -.37 (-.58, -.20) |
| *r*_D_ | -- | -1.00 (-1.00, 1.00) | | -- |  | -- | -.71 (-1.00, .04) | -- |
| *r*_C_ | 1.00 (-1.00, 1.00) | -- | | -- |  | .71 (-1.00, 1.00) | -- | -- |
| *r*_E_ | -.08 (-.25, .09) | -.09 (-.25, .09) | | -.10 (-.26, .08) |  | .07 (-.10, .22) | .07 (-.09, .22) | .07 (-.10, .22) |

*Note*. Values are estimate (95% CI). Correlations estimated as, for example, *b*_A_ / √ (*a*_a_^2^ + *b*_A_^2^).

#### Supplementary Table 11. Descriptives and Twin Correlations for Visual Search Efficiency among Included Participants

| **Variable** | ***n (*Individuals)** | **Observed Range** | ***M*** | ***SD*** | ***n* (Twin Pairs)** | | **Twin Correlations** | |
| --- | --- | --- | --- | --- | --- | --- | --- | --- |
|  |  |  |  |  | **MZ** | **DZ** | ***r*MZ** | ***r*DZ** |
| Visual Search Efficiency | 574 | -1008-2577 | 746 | 523 | 155 | 157 | .24 (.08, .40) | .01 (-.16, .18) |

*Note*. Parenthetical values are 95% confidence intervals; unit for latencies is milliseconds. Visual search efficiency calculated as the average of target-present and target-absent RT differences between the set size 8 and 28 conjunction search conditions.

#### Supplementary Table 12. Bivariate Visual Search Efficiency and IQ Model Fit Comparisons

| **Variable** | **Model** | **AIC** | **BIC** | **CFI** | **RMSEA** | **χ^2^ (*df*)** | **Comparison Model^a^** | **Δχ^2^ (*df*)** |
| --- | --- | --- | --- | --- | --- | --- | --- | --- |
| IQ and Visual Search Efficiency^b^ | 0. *ACE* | 3147.13 | 3188.37 | .99 | .02 | 18.03 (17) | -- |  |
|  | 1. *ADE* | 3145.98 | 3187.22 | 1.00 | .00 | 16.88 (17) | -- |  |
|  | **2. *AE*** | **3141.13** | **3171.13** | **1.00** | **.00** | **18.04 (20)** | **0** | 0.01 (3) |
|  |  |  |  |  |  |  | **1** | **1.16 (3)** |
|  | 2a. Drop A_vse_ | 3143.99 | 3170.23 | .99 | .02 | 22.90 (21) | 2 | 4.85* (1) |
|  | 2b. Drop *b*_A_ | 3150.53 | 3173.03 | .94 | .05 | 31.44 (22) | 2a | 8.54* (1) |

*Note*. Bolded models were best-fitting models.

^a^ Comparison model is for χ^2^ difference test of nested models.

^b^ Cross-twin, cross-trait correlations, *r*MZ = -.15 (-.25, -.04); *r*DZ = -.03 (-.15, .10). Model fit: χ^2^ (17) = 16.69, *p* = .48, CFI = 1.00, RMSEA = .00.

* *p* < .05 for χ^2^ and Δχ^2^.

#### Supplementary Table 13 Bivariate Visual Search Efficiency and IQ Model Results

|  | **Visual Search Efficiency and IQ** | | |
| --- | --- | --- | --- |
|  | ***ACE* Model** | ***ADE* Model** | ***AE* Model** |
| ***Unstandardized Parameter Estimates*** | | | |
| IQ | | | |
| *a*_IQ_ | 0.86 (0.66, 0.93) | 0.80 (0.35, 0.91) | 0.86 (0.77, 0.94) |
| *d*_IQ_ | -- | 0.31 (-0.45, 0.77) | -- |
| *c*_IQ_ | 0.10 (-0.02, 0.52) | -- | -- |
| *e*_IQ_ | 0.50 (0.43, 0.58) | 0.50 (0.43, 0.57) | 0.50 (0.43, 0.57) |
| Regression | | | |
| *b*_A_ | -0.17 (-0.37, -0.05) | 0.00 (-0.35, 0.28) | -0.17 (-0.28, -0.05) |
| *b*_D_ | -- | -0.47 (-0.59, 0.01) | -- |
| *b*_C_ | 0.06 (-0.22, 0.37) | -- | -- |
| *b*_E_ | 0.09 (-0.04, 0.22) | 0.10 (-0.04, 0.23) | 0.09 (-0.04, 0.22) |
| Residual Visual Search Efficiency | | | |
| *a*_vse_ | 0.40 (0.00, 0.56) | 0.00 (-0.30, 0.45) | 0.41 (0.05, 0.57) |
| *d*_vse_ | -- | 0.001 (-0.39, 0.48) | -- |
| *c*_vse_ | 0.00 (-0.001, 0.06) | -- | -- |
| *e*_vse_ | 0.89 (0.79, 0.99) | 0.88 (0.77, 0.97) | 0.89 (0.79, 0.99) |
| ***Implied Variance Correlations*** | | | |
| *r*_A_ | -.40 (-1.00, -.12) | .32 (-1.00, 1.00) | -.37 (-.96, -.12) |
| *r*_D_ | -- | -1.00 (-1.00, .49) | -- |
| *r*_C_ | 1.00 (-1.00, 1.00) | -- | -- |
| *r*_E_ | .10 (-.05, .25) | .12 (-.04, .25) | .10 (-.05, .25) |

*Note*. Values are estimate (95% CI). Correlations estimated as, for example, *b*_A_ / √ (*a*_vse_^2^ + *b*_A_^2^).
